# Improving neuroplasticity and Quality of Life in children with Cerebral Palsy: a customized intensive motor training protocol integrating the HABIT-ILE approach

**DOI:** 10.3389/fresc.2025.1613103

**Published:** 2025-10-13

**Authors:** Valeria Vacchini, Benedetta Brafa, Roberta Nicotra, Elena Capelli, Sabrina Signorini, Verusca Gasparroni, Arianna Michelutti, Viola Oldrati, Jessica Galli, Cosimo Urgesi, Zaira Cattaneo, Elisa Maria Fazzi, Renato Borgatti, Alessandra Finisguerra, Simona Orcesi, Boost Working Group, B. Brafa

**Affiliations:** ^1^ Unity of Child Neurology and Psychiatry, IRCCS Mondino Foundation, Pavia, Italy; ^2^ Department of Brain and Behavioral Sciences, University of Pavia, Pavia, Italy; ^3^ Scientific Institute, IRCCS E. Medea, Bosisio Parini (LC), Italy; ^4^ Department of Clinical and Experimental Sciences, University of Brescia, Brescia, Italy; ^5^ Unit of Child Neurology and Psychiatry, ASST Spedali Civili of Brescia, Brescia, Italy; ^6^ Department of Human and Social Sciences, Universitas Mercatorum, Roma, Italy; ^7^ Department of Human and Social Sciences, University of Bergamo, Bergamo, Italy

**Keywords:** neuroplasticity, children, adolescents, cerebral palsy, intensive training, HABIT-ILE, quality of life, upper limb motor deficit

## Abstract

**Introduction:**

Cerebral Palsy (CP) refers to a heterogeneous group of disorders resulting from early brain injury during development. The clinical and functional consequences are variable, but primarily characterized by motor and postural deficits that limit independence in activities of daily living, impacting child's and family's quality of life. There is consensus on the effectiveness of rehabilitative interventions when started early and administered intensively, leveraging neuronal plasticity. The Hand-Arm Bimanual Intensive Training Including Lower Extremities (HABIT-ILE) rehabilitation approach was developed to improve motor skills in children with CP, focusing on bimanual activities with integration of the lower limbs. The aim of this study is to present an intensive, individualized motor training protocol, based on HABIT-ILE principles, tailored for children and adolescents with CP.

**Methods:**

To develop the protocol, we conducted a review of literature on HABIT-ILE applications. Additionally, we carried out multidisciplinary focus groups with professionals from three Italian Centers. These discussions focused on therapeutic setting, identifying materials, structuring play activities, to define strategies to enhance applicability and impact of the protocol.

**Results:**

An intensive intervention protocol based on HABIT-ILE was developed. It consists of 30 h over 10 consecutive days, with daily sessions of 3 h. The intervention, structured around bimanual activities and lower limb involvement, was personalized according to clinical and motivational profile and conceived to be administered in pairs to children aged 6–17 years with CP and upper limb asymmetry. Sessions are divided into three components: bimanual tasks, occupational activities, and gross-motor activities, ensuring a global approach and enhancing neuroplasticity. Daily activities are selected by patients from a predetermined pool chosen by therapists, based on individual profiles and adapted progressively.

**Discussion:**

The HABIT-ILE model represents an intensive and individualized approach for improving motor abilities in these patients. Our protocol, including personalization in an ecological context and pairwork, could increase motivation, adherence, and ultimately therapy effectiveness. We plan to verify feasibility, clinical effectiveness and sustainability of this model in multicenter contexts. Ongoing trials will provide evidence of applicability and efficacy, combined with non-invasive brain stimulation (NIBS) techniques such as transcutaneous vagus nerve stimulation or transcranial alternating current stimulation.

**Clinical Trial Registration:**

ClinicalTrials.gov, Identifiers NCT06372028 and NCT06372041.

## Introduction

Cerebral palsy (CP) is a group of disorders that affects an individual's movement, posture and balance (1) caused by early brain damage during development, affecting mainly motor development and overall functional independence. Moreover, motor disorders are often accompanied by impairments in sensation, perception, intelligence, communication, behavior, epilepsy, and secondary musculoskeletal problems (2).

CP has a significant impact on the quality of life (QoL) of children and their families, affecting a child's ability to manage daily self-care tasks and participate in recreational and learning activities (3) primarily because of reduced manual abilities and gross motor function (4).

Given its impact, CP represents one of the most common causes of childhood disability. A recent systematic review and meta-analysis by McIntyre and colleagues (5) provided an updated prevalence rate of CP: the authors outlined a slight decreasing trend in pre/perinatal cases, likely due to improvements in medical care, reporting a prevalence of 1.6 cases per 1,000 live births, while post-neonatal cases remained relatively stable at 0.8 per 10,000 live births.

Considering the complex nature of these conditions and the implications in daily activities, it is necessary to develop a multidimensional rehabilitative treatment tailored to individual needs (6, 7). Current approaches include pharmacological treatments and surgical interventions, complemented by innovative rehabilitation approaches (8). A growing body of evidence highlights the importance of intensive, early, and targeted rehabilitation interventions to promote neuroplasticity during critical developmental periods and improve motor and functional outcomes, achieving long-term benefits (7–10). Among the most effective experimental approaches, goal-directed training models have shown superior efficacy compared to simple isolated movement repetitions. These models encourage children to engage in complex motor activities that integrate into daily life, thereby enhancing functional autonomy (9, 11).

In recent years, intensive motor rehabilitation protocols for children with CP have achieved notable success, particularly those focusing on upper limb function, such as Constraint-Induced Movement Therapy (CIMT) and bimanual intensive therapy, which have gained traction for their demonstrated efficacy in improving upper-limb functionality in children with unilateral CP. These therapies emphasize the necessity of high-intensity, repetitive, goal-directed practice to foster motor learning and neural adaptations (12). Systematic reviews further support the idea that interventions combining intensity, task specificity, and individualized goals are more likely to achieve meaningful improvements in motor and functional outcomes (13).

Within the most studied motor training models, a particular approach is represented by the Hand-Arm Bimanual Intensive Therapy (HABIT) model, currently integrated into clinical rehabilitation practice. Through intensive motor training, HABIT aims to improve coordination and functional use of the upper limbs (6). Given that children with unilateral CP often experience limitations not only in their upper limbs but also in their lower limbs, the HABIT model has been adapted over time to include lower-extremity training, leading to the development of Hand-Arm Bimanual Intensive Therapy Including Lower Extremities (HABIT-ILE). This approach integrates upper and lower limb exercises to improve postural control and walking abilities (14). The HABIT-ILE approach is grounded in motor learning principles and has been shown to significantly enhance motor skills across all four limbs while promoting greater functional independence (6, 15). It also leads to improvements in visuospatial skills and brain structure and connectivity, such as in corticospinal tract fibers or in the fronto-parietal network underlying motor skill learning (16–18). The development of these structures is highly dependent on activity. Structured and repetitive motor training, such as in the HABIT-ILE approach, improves synaptic refinement, strengthens appropriate connections, and facilitates motor cortex reorganization, with positive effects on brain plasticity and motor learning (19–21). By integrating bimanual coordination with lower extremity training, HABIT-ILE takes a comprehensive approach to motor rehabilitation, targeting both upper and lower limb functions to address motor control and functional limitations holistically (6).

Despite the proven efficacy of intensive motor training approaches, there remains a need for rehabilitation protocols that are both highly individualized and capable of addressing the complex motor impairments associated with CP. HABIT-ILE's unique integration of upper and lower limb training offers a promising avenue for enhancing neuroplasticity and functional outcomes. This study aims to develop a customized intensive motor training protocol inspired by the HABIT-ILE approach, specifically tailored for children with CP.

## Materials and methods

Starting from an analysis and review of the literature concerning the HABIT-ILE approach, a multidisciplinary group of healthcare professionals, including rehabilitation therapists, child neuropsychiatrists, psychologists and researchers from three Italian Centers — I.R.C.C.S. Medea in Bosisio Parini (Lecco), I.R.C.C.S. Mondino in Pavia, and ASST Spedali Civili in Brescia — conducted focus groups to define materials, settings, and play-based tasks for different severity levels of motor functional impairments.

Throughout this process, the protocol was tailored for children and adolescents diagnosed with unilateral or bilateral spastic cerebral palsy, exhibiting significant upper limb involvement and asymmetry, and aged between 6 and 17 years.

More specifically, individuals with upper limb disabilities classified at levels I-III on the Manual Ability Classification System^1^ (MACS), and associated motor deficits classified at levels I-III on the Gross Motor Function Classification System^2^ (GMFCS) will be included.

Exclusion criteria will comprise children and adolescents classified at levels IV and V on the MACS and those with associated motor deficits classified at levels IV and V on the GMFCS. Furthermore, other exclusion criteria will include subjects with severe visual impairment, defined as levels IV and V on the Visual Function Classification System^3^ (VFCS), and those with severe cognitive disabilities, defined as an Intelligence Quotient (IQ) ≤ 50. We also defined that children will be treated in pairs and matched based on demographic and clinical-functional criteria, focusing on the severity of the upper limb impairment, intellectual functioning, and/or age, meeting at least two of these three criteria. These three aspects allow clinicians to propose rehabilitative activities that are adequate to clinical features and suitable for both the patients of the pair. Moreover, during the training period, each proposed activity can be further personalized based on individual interests. Participants' upper limb motor performance will be classified using MACS, with levels ranging from low to moderate impairment (I–II) or moderate to severe (II–III). Regarding gross motor abilities, they are considered in the patients’ selection and also in the clinical assessment of included participants but they don't represent a matching variable, according to the upper limb focus in our training protocol.

## Results

The protocol, based on the principles of the HABIT-ILE model, focuses particularly on the involvement of the upper limbs in bimanual activities for patients with CP.

The rehabilitation intervention spreads over two consecutive weeks, with 5 weekly sessions, each lasting 3 h, for a total of 30 therapy hours. The training activities are led by a rehabilitation therapist (Neurodevelopmental Therapist, Occupational Therapist, or Physical Therapist), within a 1–2 therapist model, ensuring that the intervention remains personalized and goal-oriented.

The activities in the protocol are specifically designed and tailored to the clinical (MACS level) and demographic profiles of the participants, with the aim of improving treatment adherence and promoting greater engagement. This personalized approach ensures that each session is adjusted according to the individual needs of each patient, thus optimizing the overall effectiveness of the treatment. Each daily session is structured in three components: the first one involves fine motor activities, the second one focuses on daily life tasks chosen by the participants to stimulate motivation, and the third one includes gross motor activities, particularly adapted sports that promote postural control and the use of the lower limbs. These components are designed to offer a balanced and gradual approach, optimizing the benefits of rehabilitation. The rehabilitative activities designed in the study are based on principles of motor learning and problem-solving, with the patient being involved in finding strategies to reach a predefined goal. These strategies involve a complex interaction between perceptual, cognitive, and motor systems to carry out daily life activities. The repetition of actions and movements, along with a progressive increase in the complexity of tasks, stimulates mechanisms of brain plasticity, optimizing the benefits of the intervention and improving overall outcomes.

To ensure the effectiveness of the treatment and maintain motivation, it is crucial that the environment in which it takes place is ecological, highly motivating, play-conducive, comfortable, and suitable for children and adolescents. On the first day of activities, there will be a 30-minutes session for caregivers and children. This session is aimed at identifying their main interests and the main difficulties they encounter in their daily lives that have the greatest impact on their quality of life. Patients will then select activities from a pool of options proposed by the therapist and tailored to their clinical characteristics.

It starts with a focus on bimanual activities and then gradually introduces exercises for the lower limbs, which require postural control, balance, and gross motor movements. During the two weeks of treatment, the activities are modified to maintain an appropriate level of difficulty, stimulating motivation without causing frustration or dissatisfaction. Each task is designed to give the child the opportunity to achieve success, encouraging participation.

The activities are personalized based on the functional limitations of the upper limbs and differentiated according to the child's MACS level, organized in increasing order of difficulty. This approach ensures a targeted and effective treatment, improving tolerability and overall outcomes. Moreover, to maintain high motivation, daily activities can be framed within a broader theme, such as a trip around the world or a magical adventure.

The components of the three-hour daily treatment are detailed below.

### Component 1 - fine bimanual motor skills activities (50% of the daily session)

The first component of the daily session includes exercises of bimanual and fine motor tasks performed at a table, alternating complex tasks, such as cutting, with simpler repetitive movements, such as grasping an object with both hands. Depending on the patient's specific clinical characteristics and the progress observed, progressive integration of lower limb activities may occur from the early stages.

Examples of activities proposed for this session, and fully reported in Table 1, are subsequently adapted to the child's age, interests, motor functional and cognitive level, as well to the theme of the day.

**Table 1:** Examples of fine bimanual motor skills activities.

Session 1 (50% of the session) – Examples of fine bimanual motor skills activities		
MACS level I	MACS level II	MACS level III
1. Manipulating playdough and kinetic sand	1. Applying paint with stamps	1. Applying paint with sponges
2. Using finger paints	2. Manipulating playdough	2. Exploring figures covered with sensory materials, such as shaving foam
3. Assembling puzzles and dominoes	3. Manipulating kinetic sand	3. Manipulating playdough
4. Ripping pieces of crepe paper or alternative materials, forming spherical shapes and transferring them between containers with tools (hands, cups, spoon)	4. Creating handprints on paper	4. Transferring flour and similar substances between containers using big tools or plastic glasses
5. Using tongs or wooden sticks to pick up objects of different shapes, sizes, and textures (plastic or cork caps, aluminum or polystyrene balls), hidden in sand or immersed in liquid containers	5. Coloring with finger paints (initially with the whole hand, then with fingertips)	5. Assembling large puzzles and tangrams
6. Participating in activities like building with Lego bricks	6. Transferring colored liquids between containers using sponges and various tools	6. Stripping colorful paper and inserting it into designated slots
7. Reproducing models using small beads or magnets	7. Assembling medium-sized puzzles	7. Creating spheres of colored paper and gluing them to form 3D designs
8. Threading a malleable wire through a perforated board	8. Using bowls and spoons to recover objects scattered in various containers or immersed in flour or water	8. Stringing medium-sized beads onto rigid thread
9. Executing figure outlines and adhering them to cardboard supports	9. Formulating mixtures and solutions (like simulated potions) by combining various compounds	9. Performing magic tricks
10. Formulating mixtures and solutions, like simulated potions	10. Reproducing 2D and 3D models with medium-sized beads	10. Reproducing 2D and 3D models using large beads
11. Manipulating screws and bolts through rotary movements	11. Participating in card games and memory	11. Tracing pre-defined figures in flour or foam using fingers
12. Reproducing patterns with colored elastics on a pinboard	12. Cutting and gluing colorful geometric shapes to recreate complex forms, like mosaic compositions	12. Manipulating figurines immersed in a sensory bag with liquid, using digital manipulation
13. Recreating specific configurations by inserting colored elastics between fingers		
14. Participating in shanghai game		

For instance, the puzzle activity will be proposed using larger pieces for children with greater upper limb impairment, and progressively using smaller pieces for individuals with milder motor impairment. In cases of more severe limitation in upper limb use, the required task is to employ the most affected hand as a passive support to the activity, after which the task will require greater involvement of the hand in the manipulation and interlocking of the pieces.

For subjects in the lower age group, the puzzles will consist of colorful and engaging pictures and drawings, depicting animals, for example, while for older children more complex and realistic images may be employed.

Furthermore, the images to be reconstructed will be adapted to the chosen theme, for example, animals of the savannah, a seascape, or a famous monument for the world tour theme, or musical instruments for the music theme.

Another example of an adapted activity is dominoes, which can be modified into multiple themes in accordance with the child's interests and intellectual functioning, from cartoon characters, to superheroes, to syllables to form words.

According to the HABIT-ILE approach, the same task can be repeated over the course of the treatment weeks, varying the requests and the difficulty in terms of upper limb involvement, from a lower level, which involves its use as a passive stabilizer, to a gradually higher level, which encourages its more complex and active use.

Depending on the difficulties exhibited by the child, a gradual involvement of the lower limbs can already be introduced in this first session, by altering the support on which the child will be sitting during bimanual tasks, from the chair or other stable support, to the more or less inflated fitness ball, to the roller, to the balance board. The upper limb activities in which the child demonstrates more difficulty will be introduced in a stable sitting position, while those in which the child appears and is perceived as more skilled and confident will be performed under more challenging conditions that also involve the lower limbs, to an increasing extent.

During the activities, the therapist should verbalize the movement as it is performed and the goal to be achieved. He should also provide feedback on patient's performance and encourage interaction with the physical and relational environment. This helps sustaining attention and motivation, ensuring that motor, sensory, cognitive and emotional-relational skills are all engaged in building the rehabilitation project.

### Component 2 - daily-life activities (30% of the daily session)

The activities in this second component are highly variable and selected together with the child and his/her family during the initial assessment. During this part of the daily training session, indeed, participants are involved in performing activities that promote the child's autonomy, chosen based on what they have identified as relevant and challenging. These tasks are designed to stimulate motivation and respond to the child's personal needs and are expected to be easily transferable to the child's daily life.

Some examples of this component's activities are listed in the Table 2.

**Table 2:** Examples of daily-life and autonomy-related activities.

Component 2 (30% of the session)– Examples of daily-life activities	
Activity	Description
Snack preparation	Children participate in selecting ingredients and perform activities like cutting fruit, spreading jam, opening snack packages, or juicing an orange
Dressing and underdressing	Role-play activities with costumes, such as wizard, gladiator, Hawaiian, or ninja, based on the day's theme
Clothing practices	Activities involving fastening different types of closures, such as laces, zippers, bucklets, buttons and velcro
Personal hygiene	Activities like combing hair, tying hair, or brushing teeth

### Component 3 - gross motor activities (20% of the daily session)

The third component of the daily training session consists of the execution of gross motor exercises, focusing on adapted sports activities (such as basketball, tennis, and golf) progressively involving a greater engagement of the lower limbs and requiring complex postural control.

A number of sports activities were designed and adapted for this session, including basketball, tennis, golf, darts, football, archery, and, additionally, exergames.

In Table 3 are described, as examples, the steps related to adapted basketball and tennis.

**Table 3:** Examples of gross motor activities included in the training protocol.

Component 3 (20% of the session)– Examples of gross motor activities	
Activity	Description
Basketball	Passing the ball with both hands simultaneously and alternately, gradually increasing the ball size.
	Performing passes while sitting on an unstable surface (e.g., fitness ball), bouncing the ball on the ground before catching it.
	Dribbling the ball alternating the use of upper limbs.
	Performing passes while standing on a balance board.
	Managing the ball on a balance board.
	Introducing an obstacle course (hoops, cones, chairs) to pass through while dribbling.
	Shooting at the basket from a standing position.
	Attempting a jump shot at the basket.
Tennis	Bouncing the ball on the racket from a seated position.
	Hitting the ball with the racket bouncing it off the wall, first from a seated position, then from a standing position.
	Simulating a ping-pong table, hitting the ball with the racket to bounce it in the other half of the court.
	Hitting the ball with the racket to bounce it off the wall and catching it with the racket.
	Simulating a ping-pong table, hitting the ball to bounce it in the other half of the court.
	Performing passes from a standing position without support from the table, trying to move within their half of the court.

Regarding the progression of difficulty in lower limb involvement during the training sessions, we adopted the indicators proposed by the HABIT-ILE model, fully described by Bleyenheuft & Gordon (6).

For instance, if the daily theme is “trip around the world”, patients can take part in activities such as painting the flags of different countries as a bimanual activity, preparing typical meals from various cultures during an autonomy-related activity, and play a typical sport from a selected country (e.g., basketball for U.S.A. or soccer for Italy) at the end of the daily session.

The defined protocol, together with specific materials, was shared with all the participating centers to standardize training practices and mitigate potential biases associated with non-homogeneous interventions.

To improve the engagement and participation of children and families, thereby fostering adherence to the study, specialized informational materials were developed. These materials include a brochure and a video presentation, which provide a comprehensive and simplified explanation of the proposed trial and its phases.

## Discussion

Cerebral Palsy is a leading cause of childhood disability, affecting motor function and overall independence. Given its lifelong impact, early and intensive interventions are critical in maximizing motor recovery and functional potential (25, 26). Furthermore, research by Novak and colleagues (8) highlights the necessity of combining high-intensity practice with targeted functional goals to induce neural adaptation and maximize therapeutic outcomes.

In this study, we presented a structured training protocol, created in a multicentre and multidisciplinary context which follows the well-established HABIT-ILE model, originally designed for unilateral CP rehabilitation. The combination of upper and lower limb training within daily functional tasks makes HABIT-ILE as an innovative approach in pediatric CP rehabilitation. It has shown evidence in promoting improvements in whole-body functioning, by incorporating lower-limb training and bimanual coordination (6). Our intensive rehabilitation program integrates upper- and lower-limb exercises into game-based and daily functional tasks, within a motivating and ecological framework, fostering both adherence and functional improvements.

While original HABIT-ILE model was designed for 60 h intervention, recent studies (21, 27, 28) have shown that clinical meaningful goals can be also achieved with lower dose intensive treatments. Specifically, in a review by Jackman et al. (29), the authors demonstrated that interventions characterized by individualization, patient-centred goals and active participation of children require fewer hours, with individual goals achievable within 14–25 h of practice. Furthermore, according to the authors (29), a threshold dose of 30–40 h can lead to improvements in unilateral motor skills. Given these evidences, we considered the dose of 30 h as a balance, ensuring at the same time clinical improvements and also training's feasibility and sustainability. However further research is necessary to investigate and define optimal intensity levels.

A fundamental aspect of the protocol is the high level of personalization in the proposed activities: the intervention is specifically tailored to the clinical and demographic profiles of each child, ensuring that tasks align with their motor abilities and cognitive capacities. By prioritizing motivation and personalization, as recommended by most recent evidence (30) and guidelines (31), our protocol not only enhances motor function but also fosters greater independence in daily activities, ultimately improving quality of life. This dual focus aligns with evidence supporting interventions that prioritize functional goals and environmental adaptation (32). Its emphasis on motivation-driven and personalized therapy ensures broad applicability and potential scalability across different, clinical and home-based, settings. The progression of motor request through the training period is also an important element to address neuroplasticity (18) and, at the same time, to maintain high levels of motivation and challenge.

While our protocol shows promise, its implementation faces several challenges. It is essential to involve specifically-trained practitioners to ensure accurate evaluation and consistent application of therapeutic techniques, thereby improving adherence to the protocol and maintaining constancy of the intervention. Furthermore, the intensive nature of the intervention may create logistical difficulties for families, as it requires balancing therapy with everyday and academic commitments. As many authors highlight, finding an equilibrium between therapy intensity and family needs demands careful planning and adaptability (33). In this regard, it will be important to consider patients' and their parents' feedback to evaluate the study's overall feasibility.

Group training are described in scientific literature as very useful, especially in promoting motivation (30, 34, 35), but the group setting may not always be sufficiently challenging or meet the specific needs of all children (34). Considering this, we hypothesize that a pair work training could be an optimal strategy to sustain motivational aspects and, simultaneously, guarantee an appropriate personalization of the treatment itself. We also believe that sharing activites between two individuals can be an effective way to motivate and support each other, and to make an intensive and demanding programme more feasible. In this respect, the compatibility in terms of personality traits and interaction styles within each couple, emotional and competition-related aspects may play a crucial role in ensuring a positive therapeutic experience. Therapists must cultivate an engaging and dynamic environment where activities are perceived as enjoyable and rewarding, thus promoting sustained participation both individually and in group settings. During pairwork sessions, elements of rivalry or frustration may emerge, necessitating therapist-led interventions to foster collaboration and encourage positive social interactions.

The three-hour daily session length may also pose a challenge, requiring thoughtful time management to sustain children's engagement and prevent drops in attention or motivation.

Importantly, to enhance the effects on motor plasticity, the HABIT-ILE protocol may be combined with Non-Invasive Brain Stimulation (NIBS) techniques, such as transcutaneous vagus nerve stimulation or transcranial alternating current stimulation, which stimulate the motor system respectively in a bottom-up and top-down fashion (3). The potential for innovations such as NIBS and digital tools to complement HABIT-ILE is an exciting avenue for exploration. Scientific evidence outlines the need for emerging technologies that could further enhance the efficacy and accessibility of intensive rehabilitation protocols, paving the way for broader implementation and greater impact (36).

## Conclusion

In conclusion, the designed protocol represents an innovative and highly targeted approach for the treatment of children with CP, focusing on intensity, personalization, and functionality. Although the application results are beyond the scope of this study, its potential is significant, considering the growing body of evidence supporting the effectiveness of intensive and early rehabilitation interventions. We plan to verify feasibility, clinical effectiveness and sustainability of this model in multicenter contexts. The next step will be the conduction of clinical trials to validate the effectiveness of the protocol in practical settings, but the prospects for improving the quality of life for children and adolescents with CP are promising.

The continuous evolution of rehabilitation techniques, combined with more personalized approaches, could lead to a significant shift in the management of CP, enhancing not only children's motor skills but also their independence in daily activities, with a positive and lasting impact on their lives. The adoption of this protocol could become a crucial element in pediatric rehabilitation, helping to meet the needs of a growing population that faces challenges related to severe motor disabilities.

The future of rehabilitation lies at the intersection of targeted treatments, innovative technologies, and an increasing focus on the individual needs of patients and their families, paving the way for increasingly effective and accessible solutions.

## Footnotes

^1^Manual Ability Classification System (MACS) is a classification system developed to assess how children with cerebral palsy (CP) use their hands when handling objects in daily activities. It describes the child's typical manual performance rather than their maximal capacity, considering the collaborative use of both hands (22).

^2^Gross Motor Function Classification System (GMFCS) is a five-level classification system used to describe global motor function limitations in children with CP (23).

^3^Visual Function Classification System (VFCS) is a classification system to classify visual abilities of children with CP (24).

## Boost working group

Unity of Child Neurology and Psychiatry, IRCCS Mondino Foundation, Pavia, Italy: B. Brafa, R. Borgatti, A. Ciricugno, B. De Falco, R. Nicotra, S. Orcesi, S. Signorini, V. Vacchini. Department of Brain and Behavioral Sciences, University of Pavia, Pavia, Italy: R. Borgatti, E. Capelli, A. Ciricugno, R. Nicotra, S. Orcesi. Department of Clinical and Experimental Sciences, University of Brescia, Brescia, Italy: E. Fazzi, J. Galli. Unit of Child Neurology and Psychiatry, ASST Spedali Civili of Brescia, Brescia, Italy: E. Fazzi, J. Galli, A. Morandi. Scientific Institute, IRCCS E. Medea, Bosisio Parini (LC), Italy: A. Finisguerra, V. Gasparroni, C. Maghini, A. Michelutti, V. Oldrati, L. Piccinini, C. Urgesi. Department of Human and Social Sciences, Universitas Mercatorum, Roma, Italy: C. Urgesi. Department of Human and Social Sciences, University of Bergamo, Bergamo, Italy: M. Arioli, Z. Cattaneo, M. Riva.
